# Time in schizophrenia: a link between psychopathology, psychophysics and technology

**DOI:** 10.1038/s41398-022-02101-x

**Published:** 2022-08-12

**Authors:** Maria Bianca Amadeo, Davide Esposito, Andrea Escelsior, Claudio Campus, Alberto Inuggi, Beatriz Pereira Da Silva, Gianluca Serafini, Mario Amore, Monica Gori

**Affiliations:** 1 U-VIP Unit for Visually Impaired People, Fondazione Istituto Italiano di Tecnologia, Genoa, Italy; 2 Applied Neurosciences for Technological Advances in Rehabilitation Systems (ANTARES) Joint Lab: Department of Neuroscience, Rehabilitation, Ophthalmology, Genetics, Maternal and Child Health (DINOGMI), Section of Psychiatry, University of Genoa – Clinica Psichiatrica ed SPDC—Italian Institute of Technology (IIT); Largo Rosanna Benzi, 10 - 16132, Genoa, (GE) Italy; 3 Department of Informatics, Bioengineering, Robotics and Systems Engineering, Università degli Studi di Genova, Genoa, Italy; 4 IRCCS Ospedale Policlinico San Martino, Genoa, Italy

**Keywords:** Schizophrenia, Scientific community, Neuroscience

## Abstract

It has been widely demonstrated that time processing is altered in patients with schizophrenia. This perspective review delves into such temporal deficit and highlights its link to low-level sensory alterations, which are often overlooked in rehabilitation protocols for psychosis. However, if temporal impairment at the sensory level is inherent to the disease, new interventions should focus on this dimension. Beyond more traditional types of intervention, here we review the most recent digital technologies for rehabilitation and the most promising ones for sensory training. The overall aim is to synthesise existing literature on time in schizophrenia linking psychopathology, psychophysics, and technology to help future developments.

## Introduction

From the moment of their birth, newborns are immersed in time, experiencing a continuously mutating reality and creating temporal representations that naturally improve and become more sophisticated through childhood into adolescence (e.g. ref. [1–5]). Time is a fundamental element of human awareness and efficiently coding temporal proprieties of the environment is necessary to be connected with the outer world, coherently experience events, and produce adaptive behaviours.

During the last decade, research has suggested that time disturbance plays a role in the pathophysiology of schizophrenia [6–8]. However, since time is not a single, unitary dimension, literature about time representations in schizophrenia is heterogeneous, involves different terminology based on researchers’ background and does not support an easy overview and comparison between studies. The present perspective review aims at providing an overall picture of temporal representations in schizophrenia, showing that the deficit embraces time at different levels and encompasses low-level sensory alterations that must be considered when thinking of schizophrenia, its early diagnosis and its rehabilitation strategies.

## The complexity of the concept time

Time is so intrinsic in human beings that sometimes we forget how wide the concept of time is [9–16]. From a phenomenological perspective, humans are time-producing organisms, whose awareness of being is strictly interconnected with the sense of a lived duration of experience [17]. From a perceptual perspective, temporal coding is a heterogeneous process, which intervenes every time an estimation of a time duration is made but also every time sensorimotor information takes place.

To grasp the concept of time, many definitions and models have been proposed. The models developed to describe timing functions in humans can be mostly grouped into dedicated or intrinsic models [18]. On the one hand, according to dedicated models, temporal coding relies on specialized neural functions of a specific timing network. On the other hand, intrinsic models consider the timing functions as an emergent property of neural activity [19]. Thus, prototypical dedicated models of time hypothesize the existence of a centralized internal clock: [11–13] a pacemaker emits pulses, these are stored into an accumulator, then transferred to working memory, and a final decision making stage compares the pulses accumulated in working memory to those already stored in a reference memory module and identifies an appropriate outcome. Prototypical intrinsic models instead, such as the striatal beat frequency model [14–16], suggest that timing does not rely on dedicated circuits but it consists of a general computation of most neural networks [20, 21].

Specific processing circuits have been investigated for at least three time scales: sub-second, near-second, tens-of-seconds scales. Based on the main brain areas involved, it has been suggested that sub-second intervals activate an automatic timing processing (expression of the right cerebellum activity, left sensorimotor cortex and bilateral supplemental motor areas [22–24]), whereas supra-second intervals involve a cognitive timing processing (expression of the activity of right dorsolateral-prefrontal cortex and right posterior-parietal cortex [23, 25]). Although the evidence of the recruitment of different circuits between short sub-second and long supra-second intervals, the mechanisms underlying processing of near-second intervals and the neural basis for the intrinsic sense of time are still not clear [9, 10]. Moreover, time scales do not represent the only parameter to classify and investigate time processing. The next section introduces two important concepts to have in mind when investigating time.

### Distinction between perception of time and timing of perception

Traditionally, a distinction has been made between explicit and implicit level of processing of time [26, 27]. Explicit processing involves explicit judgements about external stimuli’s temporal properties, such as their duration, order or simultaneity [6]. On the contrary, implicit processing is automatically engaged whenever sensorimotor information is temporally structured, even without the specific instruction to focus on time [26, 28]. For example, it can happen that, although no overt estimates of stimulus or action duration are required, to make perceptual judgements about stimulus features or perform a specific motor act any temporal structure intrinsic in stimulus presentation or motor execution implicitly recalls timing mechanisms. Implicit and explicit processing have been dissociated with imaging methods [27]. Explicit timing engages specifically the basal ganglia, with co-activation of supplementary motor area, inferior frontal cortex and cerebellum; implicit timing activates most consistently cortical action circuits, such as left-lateralized premotor and inferior-parietal cortices.

**Fig. 1:**
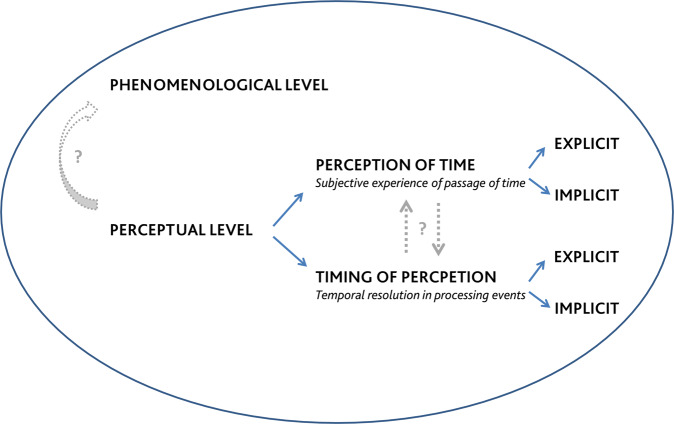
The concept of time. Time involves both a phenomenological and a perceptual level. At perceptual level, it is possible to distinguish perception of time from timing of perception, which can both be investigated trough implicit or explicit experimental paradigms.

Stated the existing difference between explicit and implicit timing, we think that, in order to shed light on the literature about time perception in schizophrenia, it is important to introduce a new distinction between two concepts, which are often confused: *perception of time* and *timing of perception*. Perception of time refers to the subjective experience of the passage of time and of events duration. Instead, timing of perception refers to the temporal resolution in processing events. Indeed, every sensory experience is embedded in time and there is, unavoidably, a time in which perceived events take place. From a phenomenological perspective, the distinction between these two concepts overlaps with the distinction between structure and content. However, it is important to clarify that the distinction between perception of time and timing perception does not necessarily coincide with explicit and implicit temporal processing respectively. As illustrated in Fig. 1, both perception of time and timing of perception can be investigated explicitly and implicitly. Some examples are presented to illustrate possible experimental paradigms that outline the difference between implicit and explicit perception of time and timing of perception:

- Explicit investigation of perception of time occurs for instance in duration discrimination tasks, where two time intervals are presented in sequence and the participant is asked to decide which interval is longer (e.g. refs. [29, 30]);
- Implicit investigation of perception of time occurs for instance in tasks that require to evaluate whether moving stimuli will collide (e.g. ref. [27]). Participants implicitly use temporal information inherent to the speed of moving sensory stimuli to predict their eventual locations.
- Explicit investigation of timing of perception occurs for instance in the simultaneity judgement task [31], which consists of evaluating the simultaneity vs. asynchrony of two stimuli. The specific instruction (“Are these stimuli simultaneous?”) makes it explicit, the possibility to estimate the timeframe within which multiple stimuli are highly likely to be perceived as one makes it a paradigm to explore timing of perception.
- Implicit investigation of timing of perception occurs for instance in the double-flash illusion task [32], in which participants are asked how many flashes they perceive, and the illusion occurs when one flash simultaneously accompanied by two beeps is erroneously perceived as two flashes. The instructions are not about time, but the number of perceived flashes indirectly gives information about the temporal resolution in processing the sensory events.

In our opinion, a categorization to better unfold the mechanisms underlying the concept of time and their role in schizophrenia would discern between perception of time and timing of perception regardless of the paradigm type, either explicit or implicit (Fig. 1).

## Time representations in schizophrenia

Schizophrenia is characterized by positive symptoms (such as disorganized thinking, speech, delusions, hallucinations), negative symptoms (such as diminished emotional expression, social withdrawal, anhedonia) and cognitive dysfunction in several domains (such as attention, working memory and executive functioning) [33]. Since Stransky and Bleuler’s classical psychopathology up to Andreasen’s Cognitive Dysmetria model, the pathogenesis of schizophrenia has been hypothesized to depend on uncoordinated activity between different brain areas [34]. The model of Cognitive Dysmetria, in particular, suggests that impaired temporal information processing may represent one core deficit that triggers a detrimental cascade of effects [8, 34–36] contributing to the positive and negative symptoms of the disease.

### Phenomenological evidence of time disturbance in schizophrenia

At phenomenological level, patients with schizophrenia report loss of continuity in the sense of time and temporal fragmentation of self-experience [37–39]. For example, they do not know the time of day, day of the week, month, or even year [40], and sometimes they feel time running slower or faster [41]. Abnormal temporal experience has been considered one key aspect of the basic self-disorder, a core trait-phenomenological feature of schizophrenia that implies structural instability of the first person-perspective [42–44]. Time, indeed, is a dynamic component of consciousness and phenomenological philosophy has interpreted the concept of time as a basic structure of the human self [45]. Fuchs and Pallagorsi [46] deeply addressed the phenomenological perspective of time in schizophrenia and concluded that “key schizophrenic symptoms such as thought disorder, thought insertion, auditory hallucinations, and passivity experiences may be described as disturbances of transcendental constitution of inner time consciousness or of the microstructure of temporality”.

Beyond the phenomenological evidence of time disturbance in schizophrenia, experimental psychology provided quantitative evidence of this temporal deficit showing that it also involves the perceptual level (see review of ref. [6, 47]). These aspects, albeit different, are likely to be interconnected (Fig. 1). Clarifying this link is important to understand the disease deeper and investigate how tailoring treatments on one ability can affect the other.

### Overview of perception of time and timing of perception in schizophrenia

Experimental psychology demonstrated that perceptual temporal processing is compromised in schizophrenia. Several reviews have extensively and successfully addressed this topic [6, 44, 47]. Here, we argue that the well-documented temporal deficit in perception encompasses both perception of time and timing of perception.

Concerning the perception of time, literature is rich of examples (for a review see ref. [6, 47]). To briefly sum up, temporal precision (i.e. the consistency in perception) is clearly impaired in patients with schizophrenia, suggesting that the patients’ explicit and implicit judgements on time are significantly more variable compared to healthy controls. Patients with schizophrenia have deficits in explicitly detecting rhythm irregularities and in estimating durations ranging from milliseconds to several minutes, in a wide range of tasks, involving both verbal and motor responses, and affecting different sensory modalities. As stated above, while the higher time-scale processing is more related to high cognitive functions, such as attention and memory, the lower time-scale processing is thought to take place automatically and at low-sensory level [6]. Selective deficits in perception of time have also been observed with implicit paradigms. For instance, schizophrenic patients have problems in using temporal cues implicitly to anticipate target occurrence during an attention orienting task (e.g. ref. [48]).

Concerning the timing of perception, low-level processing of stimuli’s temporal features has often been reported altered in schizophrenia too. For example, tasks investigating the ability to judge the simultaneity of two events have widely demonstrated an enlarged temporal binding window in patients with schizophrenia (for a review [49]). The temporal binding window is a timeframe within which multiple stimuli are highly likely to be perceived as one [50]. Extended temporal binding window indicates imprecise temporal coding of sensory stimuli. Most studies on schizophrenia reported that the temporal binding window is tens of milliseconds wider in patients with schizophrenia compared to typical adults for unisensory modalities [51–53], and it widens to hundreds of milliseconds for audiovisual [53] and audiotactile [54] stimuli. A larger audiovisual temporal binding window encompasses both semantic [55] and pure non-semantic audiovisual stimuli [56]. In addition, the patients’ difficulties in the timing of perception go beyond explicit judgements and deficits have been reported with different implicit tasks too [55–59]. For instance, Giersch et al. [28] revealed that schizophrenic patients struggle to anticipate and follow information over time through a Stimulus-Response Compatibility paradigm [60], used for the implicit evaluation of sensory information processing over time. The latter branch of studies suggests an underlying difficulty exists in how information itself is processed in time and, consequently, in processing the flow of events [44].

### Sensory processing underlying time representations at the core of schizophrenia

The excursus on time representations in schizophrenia highlights a strong deficit at the perceptual level that encompasses both the subjective experience of the passage of time (i.e. perception of time) and the ability to process temporal information intrinsic to any perceptual events (i.e. timing of perception). As a consequence, one might hypothesize that low-level sensory abnormalities underlying temporal processing are, at least partially, responsible for the overall temporal impairment and contribute to the other symptoms of schizophrenia.

To date, a growing body of literature supports such hypothesis. Indeed, overestimation of temporal intervals of both visual and auditory stimuli have been related to positive symptoms of schizophrenia (for a review see ref. [61],) and similar sensory alterations in time estimation have been observed in people without a diagnosis of schizophrenia but prone to visual hallucinations [62], individuals with schizotypal features [63] and subjects at high genetic risk for developing schizophrenia [64], suggesting that the deficit in perception of time may be an endophenotype of schizophrenia [6]. At the same time, anomalies in what we refer to as timing of perception have been proposed as a core deficit in the prodromal phase of schizophrenia [51]. For example, a larger temporal binding window is associated with disorganisation symptoms [52] and hallucination severity [53], and it matches higher levels of schizotypy in subclinical populations [54, 65, 66].

Moreover, timing of perception is crucial for multisensory integration, that is, the human ability to merge different sensory information from the same perceptual event into a unitary mental representation. The possibility and strength of multisensory integration depend on the low-level physical stimuli characteristics such as their timing (e.g. a pair of stimuli is likely to be integrated if they are closer in time) [50, 67, 68]. Research demonstrated that schizophrenia is characterized by different alterations in multisensory processing (for a review see ref. [49]). The ability to integrate sensory information has been associated with the emergence of a sense of self [69, 70] and, even more interestingly, there is a link between multisensory disintegration and self-disorders [71]. For example, integrating sensory information typically perceived as independent can cause sensory overload, alterations in sensory filters and ambiguous perceptual identity, causing a feeling of living in an incoherent world [72]. This symptomatology recalls what is observed at phenomenological level in patients with schizophrenia. In this regard, Martin et al. [44] have recently proposed that deficits in what we refer to as timing of perception could be associated with minimal self-disturbances in schizophrenic individuals. They argued that difficulties in integrating sensory information in time may affect the self because of their impact upon our ability to create single and stable representations, which would cause an uninterrupted flow of sensory inputs. In addition, Martin et al. [44] added that the impairment in timing of perception may also be responsible for the altered sense of time continuity also described at the phenomenological level in schizophrenia. Indeed, the representation of ourselves is stabilized within temporal windows and is experienced as being continuous in time. Instead, they demonstrated that, despite their enlarged temporal binding window, patients implicitly distinguish stimuli in time as controls do; however, differently from controls, they process stimuli individually rather than in sequence [57, 73, 74]. They interpreted their result as evidence of disturbed predictive coding in schizophrenia, suggesting that the ability to anticipate new events, while the focus is still on current ones, is compromised. This would lead to fragmentation in information processing, which impacts the sense of time continuity. This theory clearly supports a link between deficits in timing of perception, the temporal deficit reported at phenomenological level, and some main symptoms characterizing schizophrenia [75]. It is as if the sense of self required that sensory information were experienced as continuous in time, and in turn the sense of temporal continuity relied on the possibility to retain the latest past information and predict the imminent future events.

Therefore, research on both perception of time and timing of perception suggests that an impairment in the temporal aspects of early perceptual processing may be a pivotal substrate of psychotic functioning [76]. Starting from this perspective, major symptoms of schizophrenia, as those observed at phenomenological level, may be regarded as displays of globally disturbed time processing [46]. A recent study explored the relationship between perception of time and timing of perception and suggested that a higher precision in timing of perception implies a more fine-grained sense of the passage of time. Thus, there may be a common mechanism underlying these two perceptual dimensions [77] and its alteration may contribute to the pathophysiology of patients with schizophrenia. However, findings on the relationship between these dimensions of timing processing are inconsistent and do not allow any specific conclusion. The link between perception of time and timing of perception still needs further investigation, as well as the understanding of the interconnections between different aspects of the time deficit and psychosis. Yet, results presented in this paragraph are encouraging and, in our opinion, call clinicians and rehabilitators to consider the impairment in temporal representations at sensory level within the clinical rehabilitative framework.

## New insight for rehabilitation of schizophrenia based on sensory processing of time representations

Despite the introduction of novel pharmacological agents and psychosocial interventions, schizophrenia remains one of the most severe and debilitating mental disorders [78], affecting 1% of the population [79, 80]. Although we have seen that a perceptual impairment in time processing has been frequently described, it is still neglected at both clinical and rehabilitative level. Indeed, existing treatments address neither the overall temporal deficit nor the related sensory alterations in perception. Beyond pharmacological treatments, interventions for schizophrenic patients are mostly focused on rehabilitation of cognitive and psychosocial skills. Some examples are the Cognitive Remediation Therapy (CRT) [81], cognitive-behavioural informed psychological interventions [82], or psychosocial lifestyle interventions [83]. However, if alterations in time representation at the perceptual level are at the core of psychosis, as hypothesized in previous sections, then they should be considered during both the assessment and rehabilitation phases of schizophrenia. In addition, since there is some evidence that they characterize the prodromal phase of the disease e.g. refs. [62–64], they could become a useful tool for screening or early intervention.

Notwithstanding that further research is necessary to clarify the role of the time deficit, all kinds of task presented above could potentially complement standard screening procedures to discriminate between healthy people and patients, to assess the level of severity of the disease, or, for example, to plan targeted interventions. For rehabilitation, sensory training addressing the specific impaired temporal skills could be planned. For example, previous research demonstrated that the most reliable sense to represent time is hearing [84], and hearing calibrates, regarding the perception of time, the other sensory modalities during development [85]. Hence, intervention strategies to improve the overall impairment in perception of time in schizophrenia could target hearing. Previous research demonstrated the potential of training auditory temporal skills and showed that the learned improvements on the temporal task transferred from the auditory modality to the visual one [86]. Similarly, other studies showed the benefits of perceptual learning to improve temporal judgements (e.g. refs. [87, 88]). Concerning the timing of perception, previous studies demonstrated high plasticity of multisensory integration in time, suggesting it is sensitive to experience and it can be successfully modified through perceptual training [89, 90] and temporal recalibration [91]. Perceptual training consists of, for instance, training procedures that aim to narrow the temporal binding window width through sensory exposure [92, 93]. Instead, temporal recalibration involves the exposure to temporally asynchronous pairs of stimuli to modify the temporal weight ascribed to each sense. For example, healthy individuals immersed in a visual-leading-auditory environment for several minutes develop a higher tolerance for visual-leading-auditory asynchrony [94, 95] and this aftereffect of asynchrony exposure is maintained until new discrepant sensory information is presented [96]. In addition, musical training could be considered since musicians show a much smaller temporal binding window compared to non-musicians [97].

The above trainings are only few examples of how the temporal deficit of schizophrenia could be addressed. Sensory and perceptual trainings, temporal recalibration and musical training could be exploited as clinical treatments for temporal distortions. Clearly, further research is needed to investigate the generalizability of the benefits of sensory training on time. Specifically, we might expect that other aspects of the disease associated with impaired temporal processing [98], such as incoherent perception, language and communication dysfunctions, impaired social cognition and self-disturbance, could improve following temporal trainings at perceptual level. The next section briefly illustrates the technology developed to assess and treat schizophrenia, discussing the digital revolution’s potential for delivering perceptual training on time.

## Technology as tool for sensory intervention on time representations in schizophrenia

Nowadays, the world of e-mental Health is defined as “mental health services and information delivered or enhanced through the Internet and related technologies” [99]. This new category of technology-based assessment and intervention techniques can improve accessibility, reduce costs, ensures flexibility in terms of standardization and personalization, interactivity, and consumer engagement [100]. The potential benefits are such that various digital tools for both assessment and therapy of schizophrenia have been developed already (for reviews, see refs. [100–103]). Indeed, the disease itself does not set any specific limit to the development and use of e-mental Health-based solutions. People with schizophrenia who own digital devices do not differ from the typical population, and they have shown interest in using digital technologies for their mental health [103]. On the therapeutic side, the most popular digital interventions for schizophrenic patients include chatbots, which are software applications for monitoring signs of relapse in on-line chats, and computer-based cognitive training programs targeting different cognitive domains (e.g. CogPack) [104] or social cognition (e.g. the “Mind Reading: Interactive Guide to Emotions” [105]). These are only a few examples of technology-based interventions for psychosis; while some of them are at the very beginning, others are ready and available for clinical trials.

As the reader may have noticed, most of the efforts have been directed towards the creation of new technological ways to deliver classical therapies. However, the constant improvement of computational power and speed of digital devices [106] makes the new technologies valid tools to flexibly investigate earlier stages of sensory information processing. The study of perception requires stimuli presentation to be accurate in terms of intensity, onset, duration, and reproducibility across participants’ sessions. These characteristics are nowadays available even in smartphones, which are widely used, cheaper than standard experimental setups, and highly portable. Moreover, smartphones can deliver multimodal stimuli (i.e. visual, auditory and tactile) easily, as they can display images, animations or videos, reproduce sounds and vibrate. Thus, sensory processing underlying time representations, which requires multimodal stimulations at the millisecond scale, has become a research domain that today’s consumer electronics can probe. For instance, as described above, the deficit in the timing of perception of schizophrenic patients results in a temporal binding window around 10 to 500 ms larger than that of the typical population, depending on the task [49]. Consequently, typical paradigms on unisensory stimulation and multisensory integration require auditory, visual and/or haptic stimulations with a temporal delay among each other sometimes <20 ms e.g. ref. [107]. A few years ago, only computers with a strong GPU and a dedicated monitor with a refresh rate >100 Hz could reach such a degree of timing precision. Nowadays, as Inuggi et al. have demonstrated, consumer-level Android smartphones can deliver stimuli with the requested temporal accuracy [108, 109]. Based on their findings, Inuggi et al. developed Psysuite [108, 109], an Android app aimed to perform various psychophysics tasks that can probe, amongst others, the temporal correlates of perception. Specifically, two tasks have been validated with Psysuite: the double-flash illusion [108] and the temporal interval discrimination task [109]. The double-flash illusion task, mentioned above to illustrate the implicit investigation of timing of perception, requires inter-stimuli interval to be sometimes below few tens of milliseconds [32]. Users tested with Psysuite were subjected to this illusion. Albeit resistant to feedback training [110], the smartphone-based double-flash illusion could be used for remote assessment of temporal processing. The temporal interval discrimination task, instead, is one of the most common paradigms for explicit perception of time [111]. Participants trained in performing this task with Psysuite improved their performance after 4 days of unsupervised home training. These were just two practical examples of temporal tasks performed on smartphones, but all the above-mentioned sensory trainings on perception of time and timing of perception could be potentially delivered through new dedicated technologies. A remarkable body of literature is investigating the boundaries within which perceptual learning is obtained and generalizes to untrained stimuli [108, 109]. If temporal trainings at the perceptual level really work, they could be easily administrated during hospitalization or in the comfort of the patient’s house.

Another strength of modern digital devices yet to be fully exploited in regard to schizophrenia is immersive virtual reality. That is, the possibility to “immerse” users in virtual environments by fulfilling their senses with computer-generated visual, acoustic and haptic stimuli that mimic the perceptual features of physical stimuli and adapt in real-time to the users’ movements [112]. The power of immersive virtual reality resides in its ability to manipulate the users’ sense of embodiment [113], defined as the result of the sense of presence (i.e. self-location [114]), the sense of agency [115], and the sense of body-ownership [116]. In other words, immersive virtual reality offers a tool to investigate in a controlled and safe context the users’ sense of self, which is at the core of schizophrenia and, as hypothesized here, is likely interconnected with temporal processing. The implementation of real-time interactions with users allows to manipulate the temporal relationships between human predictions, actions, and sensory feedbacks, thus changing the sensorimotor contingencies and investigating the link between time and sense of self. Using virtual reality, it becomes possible, for instance, to control temporal delays of sensory feedback (e.g. delaying the incoming visual information), to create new sensory consequences for specific motor actions (e.g. bouncing a virtual ball by pressing a button), or to manipulate the temporal delay of sensory consequences (e.g. delaying the bounce of a virtual ball after pressing a button) within an ecological environment. All these possible experimental manipulations would allow to investigate sensory-motor temporal predictions and recalibration [117]. A link between the sense of agency in schizophrenia and imprecise motor predictions has already been identified thanks to virtual reality’s ability to on-line change the relationship between movements and corresponding sensory consequences [118–121]. Furthermore, since the human sensory-motor system seems able to adapt to temporal manipulations of the sensory-motor contingencies (see ref. [122] for a review), immersive virtual reality may offer a way to train the perception of time or the timing of perception and simultaneously assess the training effect on the sense of self [123, 124]. That said, despite the potential of such technology, to date virtual reality has been employed mainly to enhance and extend psychosocial and cognitive-behavioural treatments [125].

To sum up, the emergence of the e-mental Health has extensively expanded clinical possibilities by providing digital tools to modernise standard techniques. Digital technologies represent the most promising field of application for perceptual trainings. Considering results about sensory processing underlying time representations in schizophrenia, the scientific community should exploit the potentials of technology to create new science-based assessment and rehabilitation procedures working at sensory and sensory-motor levels. It is not about reinventing wheels; it is about using wings.

## Conclusion

In this perspective review we provided a brief overview of the existing literature showing impaired ability to process temporal proprieties of the environment in people affected by schizophrenia. By distinguishing the phenomenological from the perceptual level and, within the latter, perception of time from timing of perception, we highlighted that the temporal deficit is multifaceted and encompasses low-level sensory alterations. However, these are not considered when thinking of rehabilitation strategies and technological devices. Although various reviews have stressed out the potential of digital tools on both assessment and therapy of schizophrenia, we think that the growth of the e-mental Health services must be expanded to the range of the possible assessment, early intervention and rehabilitation techniques based on scientific evidence. Digital technologies offer powerful resources for evaluating the soft signs of the disease, giving clinicians the possibility to dig into the lower levels of sensory processing with unprecedented ease. These potentials should be considered to develop new solutions that emerge from an interdisciplinary approach, involving experts on psychopathology, psychophysics and technology.
